# Ancient feeding ecology inferred from stable isotopic evidence from fossil horses in South America over the past 3 Ma

**DOI:** 10.1186/1472-6785-11-15

**Published:** 2011-06-14

**Authors:** José L Prado, Begoña Sánchez, María T Alberdi

**Affiliations:** 1 INCUAPA, Universidad Nacional del Centro. Del Valle 5737. B7400JWI Olavarría, Argentina; 2 Museo Nacional de Ciencias Naturales, CSIC. José Gutiérrez Abascal, 2. 28006-Madrid, Spain

**Keywords:** Stable Isotopes, C_4 _plants, Mammals, horses, South America

## Abstract

**Background:**

Stable isotope ratios (^13^C/^12^C and ^18^O/^16^O) in fossil teeth and bone provide key archives for understanding the ecology of extinct horses during the Plio-Pleistocene in South America; however, what happened in areas of sympatry between *Equus (Amerhippus) *and *Hippidion *is less understood.

**Results:**

Here, we use stable carbon and oxygen isotopes preserved in 67 fossil tooth and bone samples for seven species of horses from 25 different localities to document the magnitude of the dietary shifts of horses and ancient floral change during the Plio-Pleistocene. Dietary reconstructions inferred from stable isotopes of both genera of horses present in South America document dietary separation and environmental changes in ancient ecosystems, including C_3_/C_4 _transitions. Stable isotope data demonstrate changes in C_4 _grass consumption, inter-species dietary partitioning and variation in isotopic niche breadth of mixed feeders with latitudinal gradient.

**Conclusions:**

The data for *Hippidion *indicate a preference varying from C_3 _plants to mixed C_3_-C_4 _plants in their diet. *Equus (Amerhippus) *shows three different patterns of dietary partitioning *Equus *(*A*.) *neogeus *from the province of Buenos Aires indicate a preference for C_3 _plants in the diet. *Equus *(*A*.) *andium *from Ecuador and *Equus *(*A*.) *insulatus *from Bolivia show a preference for to a diet of mixed C_3_-C_4 _plants, while *Equus *(*A*.) *santaeelenae *from La Carolina (sea level of Ecuador) and Brazil are mostly C_4 _feeders. These results confirm that ancient feeding ecology cannot always be inferred from dental morphology. While the carbon isotope composition of horses skeletal material decreased as latitude increased, we found evidence of boundary between a mixed C_3_/C_4 _diet signal and a pure C_4 _signal around 32° S and a change from a mixed diet signal to an exclusively C_3 _signal around 35°S.

We found that the horses living at high altitudes and at low to middle latitude still have a C_4 _component in their diet, except the specimens from 4000 m, which have a pure C_3 _diet. The change in altitudinal vegetation gradients during the Pleistocene is one of several possibilities to explain the C_4 _dietary component in horses living at high altitudes. Other alternative explanations imply that the horses fed partially at lower altitudes.

## Background

**Figure 1:**
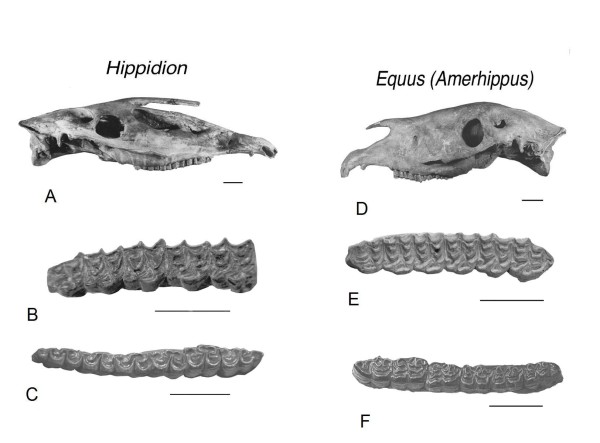
**Skull and dental characteristics**. A, *Hippidion *with nasal notch posterior to M_1_. B, *Equus *(*Amerhippus*). C, comparative morphology of upper cheek teeth in occlusal view.

In South America, horses are represented by two groups: equidiforms and hippidiforms. *Hippidion *are characterized by a retracted nasal notch which has been interpreted as an adaptation to the presence of a proboscis and limbs with robust metapodials. The upper teeth present an elongate-oval protocone with simple enamel plication and lower teeth have a deep ectoflexid, penetrating the isthmus (Figure 1). On the other hand, in *Equus *(*Amerhippus*) a retracted nasal notch is not present and the metapodials are slender. The upper teeth present a triangular protocone and multiple internal posfossette plications. There are features that are common to both of these groups, such as differentiation into horses of both small and large body size, which are possibly a consequence of convergence due to adaptation to similar environments. The three species of hippidiforms are included within the genus *Hippidion *Owen, 1869 [1,2] whereas the five species of equidiforms are included in the subgenus *Equus *(*Amerhippus*) [3]. Each of these groups has different ecological adaptations that are evident in the cranial morphology, robustness of the limbs and body size [4,5].

A technique that has proven useful for investigating the ecology of fossil horses is through examination of stable isotope values found in teeth and bone in combination with dental wear [6-16]. Isotopic analyses can reveal information about resource use and resource partitioning among species and is also able to determine diet and habitat use [15,17]. Here, we will address: (1) whether stable isotope values permit identification of resource use and partitioning among horse species and (2) if resource use and partitioning are determined, do the results support the ecology predicted by morphology or body size? Finally, we compare carbon isotope values and evaluate the hypothesis that dietary niches, inferred from the mean and variation of carbon isotope values, did not change throughout time in the same latitude.

Stable carbon and oxygen isotopes are incorporated into the tooth and bone apatite of horses and are representative, respectively, of the food and water consumed while alive. The carbon isotope ratio is influenced by the type of plant material ingested, which is in turn influenced by the photosynthetic pathway utilized by the plants. During photosynthesis, C_3 _plants in terrestrial ecosystems (trees, bushes, shrubs, forbs, and high altitude and high latitude grasses) discriminate more markedly against the heavy ^13^C isotope during fixation of CO_2 _than C_4 _plants (tropical grasses and sedges). Thus C_3 _and C_4 _plants have distinct δ^13^C values. C_3 _plants usually have δ^13^C values of -30 per mil (‰) to -22‰, with an average of approximately -26‰, whereas C_4 _plants have δ^13^C values of -14 to -10‰, with an average of about -12‰ [18-22]. Animals incorporate carbon isotopes from food into their teeth and bone with an additional fractionation of ~12 to 14‰ [23,24]. Mammals feeding on C_3 _plants characteristically have δ^13^C values between -14 and -8‰, while animals that eat C_4 _tropical grasses have δ^13^C values between +2 and -2‰. A mixed-feeder would fall somewhere in between these two extremes [25,26]. Hence, the relative proportions of C_3 _and C_4 _vegetation in the diet of an animal can be determined by analyzing the δ^13 ^C value of its teeth and bones.

A number of previous studies have used the carbon and oxygen isotopic abundance of fossils and paleosols from South America to reconstruct the diets of extinct herbivores and the paleoenvironmental parameters of ancient terrestrial communities and ecosystems [27-32]. Carbon isotope data for horses from South America have been presented in several papers [8,33-35]. In 1999, MacFadden et al. [36] presented the ancient distributions and latitudinal gradients of C_3 _and C_4 _grasses based on isotopic data from New World Pleistocene horses. In addition, some papers have investigated the application of geochemical techniques in conjunction with morphological and dental wear data to reconstruct the feeding ecology and niche characterization of individual species [34,36,37].

**Figure 2:**
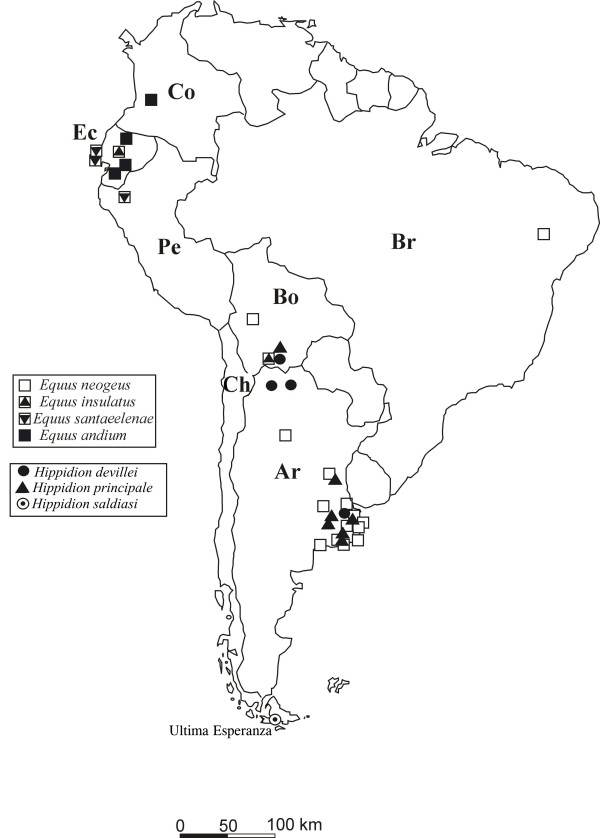
**Geographical distribution of analyzed samples**. Argentina (Ar), Bolivia (Bo), Brazil (Br), Chile (Ch), and Ecuador (Ec).

All equid taxa from South America were sampled for teeth (n:29) and bone (n:38) stable carbon and oxygen isotopes (table 1 and 2). Additional data of thirty samples were taken from MacFadden et al. [36]. Together the data represent five species within the subgenus *Equus *(*Amerhippus*): *E*. (*A*.) *andium*, *E*. (*A*.) *insulatus*, *E*. (*A*.) *neogeus*, *E*. (*A*.) *santaeelenae *and *E*. (*A*.) *lasallei *[3]. The genus *Hippidion *includes three species: *H. principale*, *H. devillei *and *H. saldiasi *[2]. Some of these valid species have a wide geographical distribution whilst others, such as *E*. (*A*.) *andium *and *E*. (*A*.) *insulatus*, are restricted to the Andes region. In contrast, *E*. (*A*.) *neogeus*, *E*. (*A*.) *santaeelenae *and *E*. (*A*.) *lasallei *are found in the non-Andean tropical or subtropical regions of South America such as Argentina, Uruguay, Colombia, Brazil, and the coastal area of Ecuador (Figure 2). *H. saldiasi *is restricted to a particular habitat in southern Patagonia [38,39]; while *H. principale *and *H. devillei *come from different localities in South America, such as Tarija in Bolivia and the Pampa region in Argentina, that cover a broad range of altitudes from 10 to 4000 m. One restriction to our study is the chronological control of the sample. Most of the samples were collected from old museum collections in Ecuador, Bolivia and Argentina. These old collections were recovered without sufficient stratigraphic control. Anyway we considered this limitation may condition the interpretations about altitudinal and latitudinal gradients for fossil samples, but do not invalidate the suggested patterns.

**Table 1:** Values of δ^18^O and δ^13^C of South American fossil *Hippidion*.

Species name (age)	Specimen	Skeletal	Altitude	Locality (country)	Latitude	d^13^C (CO_3_)	d^18^O (PO_4_)	d^18^O (CO_3_)
	number	tissue	m.asl			‰ PDB	‰ V-SMOW	‰ V-SMOW
*H. devillei *(44 to 21 ka BP)	MACN 1621	b	1866	Tarija (Bo)	22S	-8.8	19.7	26.1
*H. devillei *(44 to 21 ka BP)	MACN 1516	t	1866	Tarija (Bo)	22S	-10.8	19.3	28.7
*"O". devillei *(44 to 21 ka BP)^1^		e	1866	Tarija (Bo)	22S	-6.6		
*H.principale *(44 to 21 ka BP)^1^		e	1866	Tarija (Bo)	22S	-10.3		
*H.principale *(44 to 21 ka BP)^2^		e	1866	Tarija (Bo)	22S	-8.8		
*H. devillei *(12.8 to 11.5 ka BP)	MLP 85-VII-1-2	t	4000	Mina Aguilar (Ar)	23S	-9.8	17.2	24.1
*H. devillei *(12.8 to 11.5 ka BP)	MLP 85-VII-1-4	b	4000	Mina Aguilar (Ar)	23S	-10.0	14.2	25.2
*H. devillei *(Late Plio)	MACN 5361	b	4000	Uquía (Ar)	23S	-10.1	16.6	25.6
*H. devillei *(Late Plio)	MACN 5361	t	4000	Uquía (Ar)	23S	-10.2	17.4	25.2
*H. devillei *(Late Plio)	MACN 5364	t	4000	Uquía (Ar)	23S	-9.9	16.8	25.3
*H. devillei *(Late Plio)	MACN 5364	b	4000	Uquía (Ar)	23S	-10.3	16.0	25.9
*H. devillei *(Early Pl)	MACN 2140	t	10	Olivos (Ar)	35S	-9.8	21.5	29.8
*H. devillei *(Early Pl)	MACN 2138	t	10	Olivos (Ar)	35S	-11.7	20.2	28.8
*H. devillei *(Early Pl)	MACN 2155	b	10	Olivos (Ar)	35S	-10.2	19.7	27.7
*H. principale *(Middle Pl)	MLP 6-19	t	10	Paraná (Ar)	32S	-8.4	19.4	28.9
*H. principale *(Middle Pl)	MLP 6-19	b	10	Paraná (Ar)	32S	-8.7	19.1	29.3
*H. principale *(Early Pl)^3^	MLP 6-441	e	10	Buenos Aires (Ar)	35S	-12.9		
*H. principale *(Early Pl)^3^	MLP 6-435	e	10	Buenos Aires (Ar)	35S	-10.2		
*H. principale *(28 to 10 ka BP)	MACN 10441	e	10	Luján (Ar)	35S	-10.6	21.2	30.2
*H. principale *(28 to 10 ka BP)	MACN 10441	d	10	Luján (Ar)	35S	-12.1	20.9	30.7
*H. principale *(28 to 10 ka BP)	MLP 6-364	b	10	Luján (Ar)	35S	-11.3	19.5	30.6
*H. principale *(28 to 10 ka BP)	MACN 6092	b	10	Luján (Ar)	35S	-11.2	21.7	30.5
*H. principale *(28 to 10 ka BP)	MACN 5667	t	10	Río Salado (Ar)	36S	-12.0	19.8	28.8
*H. principale *(28 to 10 ka BP)	MACN 5667	b	10	Río Salado (Ar)	36S	-10.1	19.9	28.3
*H. principale *(28 to 10 ka BP)	MACN 5056	b	10	Río Salado (Ar)	36S	-8.2	20.2	30.1
*H. principale *(28 to 10 ka BP)	MACN 1735	t	50	Arroyo Tapalqué (Ar)	38S	-9.8	20.2	29.2
*H. principale *(28 to 10 ka BP)	MACN 5265	b	50	Arroyo Tapalqué (Ar)	38S	-9.6	20.1	29.1
*H. principale *(28 to 10 ka BP)	MACN 1734	t	50	Arroyo Tapalqué (Ar)	38S	-9.4	22.1	31.5
*H. principale *(28 to 10 ka BP)	s/s	b	100	Arroyo Tapalqué (Ar)	38S	-11.6	19.5	30.8
*H. principale *(28 to 10 ka BP)	MACN 9739	b	100	Río Quequén Salado(Ar)	38S	-8.1	21.2	29.9
*H. saldiasi *(12.8 to 11.5 ka BP)	MLP 81-VI-28-5	b	200	U. Esperanza (Ch)	52S	-12.2	17.65	24.9

Samples with the same "specimen number" correspond to the same individuals. The bones correspond to the mandibular or maxillary remains that contained the teeth that were analyzed. Plio = Pliocene; Pl = Pleistocene; e = enamel; d = dentine; b = bone; t = tooth (enamel + dentine). Ar = Argentina; Bo = Bolivia; Br = Brazil; Ch = Chile. A = Andean; PL = Plain landscape. ^***1 ***^Taken from MacFadden et al. [22], ^***2 ***^taken from MacFadden and Shockey [27] and ^***3 ***^taken from MacFadden et al. [20].

**Table 2:** Values of δ^18^O and δ^13^C of South American fossil *Equus *(*Amerhippus*).

Species name (age)	Specimen	Skeletal	Altitude	Locality (country)	Latitude	d^13^C (CO_3_)	d^18^O (PO_4_)	d^18^O (CO_3_)
	number	tissue	**m.asl**.			‰ PDB	‰ V-SMOW	‰ V-SMOW
*Equus *sp. (Late Pl)*	UCMP.38100	e	440	La Venta (Co)	3N	-0.7		29.9
*Equus *sp. (Late Pl)*	ING 184268	e	440	La Venta (Co)	3N	-2.2		32.4
*E. insulatus *(Middle Pl)	V.543	b	2500	Rio Chiche (Ec)	0	-5.7	13.6	23.5
*E. insulatus *(Middle Pl)	V.542	t	2500	Rio Chiche (Ec)	0	-4.8	16.7	25.1
*E. insulatus *(Middle Pl)	V.542	b	2500	Rio Chiche (Ec)	0	-3.2	15.7	24.6
*E. insulatus *(Middle Pl)	V.544	b	2500	Rio Chiche (Ec)	0	-5.8	15.5	24.3
*E. andium *(26 to 19 ka BP)	V.455	b	2778	Alangasí (Ec)	0	-5.5	15.0	24.4
*E. andium *(26 to 19 ka BP)	V.430	b	2778	Alangasí (Ec)	0	-6.9	13.1	23.3
*E. andium *(26 to 19 ka BP)	V.4014	b	2778	Alangasí (Ec)	0	-6.3	14.8	23.0
*E. andium *(26 to 19 ka BP)	V.2495	b	2778	Quebrada Colorada (Ec)	2S	-6.2	14.3	23.9
*E. andium *(26 to 19 ka BP)	V.2417	b	2778	Quebrada Colorada (Ec)	2S	-6.7	11.3	22.0
*E. andium *(26 to 19 ka BP)	V.2163	b	2778	Quebrada Colorada (Ec)	2S	-5.8	13.2	21.7
*E. andium *(20 to 12 ka BP)	V.2161	b	2778	Punín (Ec)	2S	-9.9	14.3	25.2
*E. andium *(20 to 12 ka BP)	V.2152	b	2778	Punín (Ec)	2S	-5.8	11.4	23.8
*E. andium *(20 to 12 ka BP)	V.465	b	2778	Punín (Ec)	2S	-6.1	14.9	23.6
*E. santaelenae *(12 to 8 ka BP)	V.3037	d	0	La Carolina (Ec)	2S	9.2	22.1	34.9
*E. santaelenae *(12 to 8 ka BP)	V.3037	e	0	La Carolina (Ec)	2S	1.5	22.1	32.6
*E. santaelenae *(12 to 8 ka BP)	V.3037	b	0	La Carolina (Ec)	2S	6.1	21.8	33.5
*E. santaelenae *(12 to 8 ka BP)	V.69	b	0	La Carolina (Ec)	2S	-1.5	21.3	32.0
*E. santaelenae *(12 to 8 ka BP)*	V-3037	e	0	La Carolina (Ec)	2S	1.8		32.7
*E. santaelenae *(12 to 8 ka BP)*	V-68	e	0	La Carolina (Ec)	2S	5.4		33.6
*E. santaelenae *(26 to 19 ka BP)*	F:AM 131869	e	0	Salinas Oil Field (Ec)	3S	-0.8		32.6
*E. santaelenae *(26 to 19 ka BP)*	F:AM 131868	e	0	Salinas Oil Field (Ec)	3S	3.0		32.5
*E. santaelenae *(26 to 19 ka BP)*	ROM.3471C	e	85	Talara Tar Pit (Peru)	5S	2.1		27.0
*E. neogeus*(26 to 19 ka BP)*	uncatalogued	e	200	Ourolandia (Br)	12S	1.1		29.5
*E. neogeus*(26 to 19 ka BP)*	uncatalogued	e	200	Ourolandia (Br)	12S	1.7		30.6
*E. neogeus*(28 to 10 ka BP)*	UF.uncatalogued	e	500	Naupua-3 (Bo)	21S	0.2		31.1
*E. neogeus*(28 to 10 ka BP)*	UF.uncatalogued	e	500	Naupua-1 (Bo)	21S	0.1		29.8
*E. insulatus *(44 to 21 ka BP)	MACN 1501A	b	1866	Tarija (Bo)	22S	-5.8	17.4	25.6
*E. insulatus *(44 to 21 ka BP)	MACN 1501B	t	1866	Tarija (Bo)	22S	-5.0	20.9	26.2
*E. insulatus *(44 to 21 ka BP)	MACN 1509	t	1866	Tarija (Bo)	22S	-8.3	20.0	28.3
*E. insulatus *(44 to 21 ka BP)*	UF.uncatalogued	e	1866	Tarija (Bo)	22S	-4.8		25.2
*E. insulatus *(44 to 21 ka BP)*	UF.uncatalogued	e	1866	Tarija (Bo)	22S	-2.9		24.4
*E. insulatus *(44 to 21 ka BP)*	UF.uncatalogued	e	1866	Tarija (Bo)	22S	-2.6		25.8
*E. insulatus *(44 to 21 ka BP)*	UF.uncatalogued	e	1866	Tarija (Bo)	22S	-2.3		26.4
*E. insulatus *(44 to 21 ka BP)*	UF.90750	e	1866	Tarija (Bo)	22S	-2.9		26.2
*E. insulatus *(44 to 21 ka BP)*	UF.90895	e	1866	Tarija (Bo)	22S	-3.5		23.5
*E. insulatus *(44 to 21 ka BP)*	UF.90653	e	1866	Tarija (Bo)	22S	-4.1		26.8
*E. insulatus *(44 to 21 ka BP)*	UF.90764	e	1866	Tarija (Bo)	22S	-4.1		24.2
*E. insulatus *(44 to 21 ka BP)*	UF.91972	e	1866	Tarija (Bo)	22S	-2.7		24.9
*E. neogeus *(28 to 10 ka BP)	MCNAM-PV-83	e	200	La Banda (Ar)	28S	-1.0		28.0
*E. neogeus*(Late Pl)*	MLP.52.IX.29-91	e	10	Esperanza, Santa Fé (Ar)	32S	-0.8		30.8
*E. neogeus*(Middle Pl)*	AMNH.11154	e	10	Buenos Aires (Ar)	35S	-10.7		30.8
*E. neogeus*(Middle Pl)*	AMNH.11154	e	10	Buenos Aires (Ar)	35S	-10.3		30.6
*E. neogeus*(Middle Pl)*	MLP.91.VI.5-1	e	50	Magdalena (Ar)	35S	-10.6		30.3
*E. neogeus *(28 to 10 ka BP)	MACN 11636	b	10	Luján (Ar)	35S	-11.7	21.2	30.4
*E. neogeus *(28 to 10 ka BP)	MLP 6-402	b	10	Luján (Ar)	35S	-11.2	20.1	30.6
*E. neogeus *(Middle Pl)	MLP s/s	t	10	Cant. Vial. Prov.(Ar)	35S	-7.5	21.2	30.5
*E. neogeus *(Middle Pl)	MLP s/s	b	10	Cant. Vial. Prov.(Ar)	35S	-11.5	20.8	32.1
*E. neogeus *(28 to 10 ka BP)	MACN 6116	t	50	Arroyo Tapalqué (Ar)	38S	-8.5	17.3	29.8
*E. neogeus *(28 to 10 ka BP)	MACN 6116	b	50	Arroyo Tapalqué (Ar)	38S	-7.9	21.2	29.7
*E. neogeus *(28 to 10 ka BP)	MACN 9753	e	100	Río Quequén Salado (Ar)	38S	-9.9	21.3	30.5
*E. neogeus *(28 to 10 ka BP)	MACN 9753	d	100	Río Quequén Salado (Ar)	38S	-10.1	21.3	30.3
*E. neogeus *(28 to 10 ka BP)	MACN 9753	b	100	Río Quequén Salado (Ar)	38S	-8.0	20.4	29.8
*E. neogeus *(28 to 10 ka BP)	MACN 9751	b	100	Río Quequén Salado (Ar)	38S	-7.8	21.0	30.9
*E. neogeus *(28 to 10 ka BP)	MACN 9751	d	100	Río Quequén Salado (Ar)	38S	-8.3	22.9	31.4
*E. neogeus *(28 to 10 ka BP)	MLP 63-VI-1017	b	100	Río Quequén Salado (Ar)	38S	-9.0	20.7	30.3
*E. neogeus*(12.8 to 11.5 ka BP)*	MLP.52.X.5-3	e	100	Rio Quequén Salado (Ar)	38S	-8.5		30.7
*E. neogeus*(12.8 to 11.5 ka BP)*	MLP.52.X.5-3	e	100	Rio Quequén Salado (Ar)	38S	-7.8		30.9
*E. neogeus *(28 to 10 ka BP)	MLP s/s	t	10	P. Hermengo (Ar)	38S	-7.2	20.1	29.7
*E. neogeus *(28 to 10 ka BP)	MLP 80-VIII-13-4	b	10	Paso Otero (Ar)	38S	-10.7	20.1	29.8
*E. neogeus *(28 to 10 ka BP)	MLP 80-VIII-13-63	b	10	Paso Otero (Ar)	38S	-11.0	18.4	29.8
*E. neogeus *(28 to 10 ka BP)	MLP.80.VIII.13-93	e	10	Paso Otero (Ar)	38S	-10.2		31.3
*E. neogeus *(around 130 ka)	MLP s/s	t	10	Centinela Mar (Ar)	38S	-10.0	19.1	30.3
*E. neogeus *(12.8 to 11.5 ka BP)	MACN 14417	d	10	Zanjón Seco (Ar)	38S	-11.4	20.3	29.3
*E. neogeus *(12.8 to 11.5 ka BP)	MACN 14417	t	10	Zanjón Seco (Ar)	38S	-10.4	21.1	29.1
*E. neogeus *(12.8 to 11.5 ka BP)	MACN 14417	e	10	Zanjón Seco (Ar)	38S	-10.5	21.2	30.7
*E. neogeus *(12.8 to 11.5 ka BP)	MACN 14417	b	10	Zanjón Seco (Ar)	38S	-10.9	19.9	29.7

Samples with the same "specimen number" correspond to the same individuals. The bones correspond to the mandibular or maxillary remains that contained the teeth that were analyzed. Abbreviations as in Table 1. * taken from MacFadden et al. [25].

In order to demonstrate how dietary resources were partitioned we divided the samples into 10 different groups taking into account the genus, as well as the age and the altitude of the corresponding deposit. Data for these groups and descriptive statistics are listed in table 3: (A) all *Hippidion*; (B) all *Equus *(*Amerhippus*); (C) *Hippidion *from the Late Pleistocene; (D) *Hippidion *from the Late Pliocene to Early Pleistocene; (E) *Equus *(*Amerhippus*) from the Late Pleistocene; (F) *Equus *(*Amerhippus*) from the Middle Pleistocene; (G) *Equus *(*Amerhippus*) from the plains; (H) *Equus *(*Amerhippus*) from the mountain corridor; (I) *Hippidion *from the plains; and (J) *Hippidion *from the mountain corridor.

**Table 3:** Descriptive statistics for the eight groups of South American equids that were compared.

Groups	*n*	Mean δ^13^C	SD	Range	*n*	Mean δ^18^O	SD	Range
		(‰)PDB	(‰)	(‰)		(‰)V-SMOW	(‰)	(‰)
A	31	-10.1	1.39	-12.9 to -6.6	26	28.3	2.20	24.2 to 31.5
B	68	-5.2	4.83	-11.66 to 9.21	68	28.5	3.32	21.7 to 34.9
C	17	-10.2	1.39	-12.22 to -8.08	17	29.0	2.17	24.2 to 31.5
D	14	-10.0	1.44	-12.9 to -6.6	9	27.0	1.74	25.3 to 29.8
E	52	-5.5	5.43	-11.66 to 9.21	50	29.4	3.17	21.7 to 34.9
F	16	-4.3	1.61	-8.31 to -2.3	16	25.3	1.28	23.5 to 28.3
G	43	-5.3	5.93	-11.66 to 9.21	41	30.7	1.48	27 to 34.9
H	25	-5.1	1.85	-9.86 to -2.3	25	24.6	1.51	21.7 to 28.3
I	12	-9.8	1.34	-12.22 to -6.6	8	25.8	1.30	25.8 to28.7
J	19	-10.3	1.42	-8.08 to -12.90	17	29.7	1.00	29.7 to 31.5

A: all *Hippidion *specimens; B: all *Equus *(*Amerhippus*) specimens; C: *Hippidion *from the Late Pleistocene; D: *Hippidion *from the Late Pliocene to Early Pleistocene; E: *Equus *(*Amerhippus*) from the Late Pleistocene; F: *Equus *(*Amerhippus*) from the Middle Pleistocene; G: *Equus *(*Amerhippus*) from the plains landscape; H: *Equus *(*Amerhippus*) from the mountain corridor; I: *Hippidion *from the plains landscape and J: *Hippidion *from the mountain corridor. ***n***: number of samples. **SD**: standard deviation.

## Methods

### Materials

Fossil samples were collected from specimens stored at the following institutions in Argentina: Museo de La Plata, Museo Argentino de Ciencias Naturales "Bernardino Rivadavia" in Buenos Aires and Museo de Ciencias Naturales y Antropológicas "Juan Cornelio Moyano"in Mendoza. Museo de la Escuela Politécnica Nacional of Quito, in Ecuador. The museum specimen number, locality, country, age, skeletal tissue (enamel, bone and dentine) and the altitudinal and latitudinal distribution of each sample are given in Tables 1 and 2. We analyzed 26 samples of *Hippidion *and 41 carbon and oxygen isotope composition samples of *Equus (Amerhippus)*. Additional 32 samples values (Table 1 and 2) were taken from MacFadden et al. [31,33,36] and MacFadden and Shockey [34].

### Pre-treatment of the samples

The samples were finely ground in an agate mortar. The chemical pre-treatment of the samples was carried out as described in Koch et al. [58] in order to eliminate secondary carbonate. About 40-50 mg of powdered enamel and bone samples were soaked in 2% NaOCl for three days at room temperature to oxidize organic matter. Residues were rinsed and centrifuged five times with de-ionized water, and then treated with buffered 1M acetic acid for one day to remove diagenetic carbonates. Pre-treatment of the enamel was slightly different because samples were soaked in 2% NaOCl for only one day.

### Analysis of the samples

Carbon dioxide was obtained by reacting about 40-50 mg of the treated powder with 100% H_3_PO_4 _for five hours at 50°C. The carbon dioxide was then isolated cryogenically in a vacuum line. Results are reported as δ = ([R_sample_/R_standard_]-1 ) × 1000, where R = ^13^C/^12^C or ^18^O/^16^O, and the standards are PDB for carbon and V-SMOW for oxygen. We have applied the data corrections for calcite from Koch et al. [59] to calculate the magnitude of the oxygen isotopic fractionation between apatite CO_2 _and H_3_PO_4 _at 50°C. The analytical variation for repeated analyses was 0,1‰ for δ^13^C and 0,2‰ for δ^18^O. For the analysis of phosphate we followed the chemical treatment procedure described by Tudge [60], which resulted in the precipitation of the phosphate ions in the form of BiPO_4_. CO_2 _was obtained by reacting BiPO_4 _with BrF_5 _as described by Longinelli [61]. All the samples were run in duplicate and the reported results are the mean of at least two consistent results. The analytical precision for repeated analyses was 0,2‰.

We performed both parametric (*t*-test) and nonparametric (Wilcoxon Signed-Rank) statistical tests to evaluate δ^13^C and δ^18^O differences in Middle and Late Pleistocene populations. SPSS 15.0 software was used for the statistical analysis.

## Results

### Preservation state of the enamel, dentine and bone in the specimens analyzed

We checked the diagenetic alteration between the different skeletal tissues under the assumption that primary values were similar between different skeletal tissues from one individual. The phosphate oxygen isotope composition is usually considered to be more robust against diagenetic alteration than the carbonate oxygen at least if no bacteria are involved during the alteration processes. We measured both δ^18^O _CO3 _and δ^18^O _PO4 _values on the same specimens and obtained a regression between both, and compared those with known isotope equilibrium-relationships for biogenic apatite of modern bones and teeth [40,41]. If samples fall in the range of modern biogenic apatite this would argue for a preservation of primary δ^18^O _CO3 _and δ^18^O _PO4 _values, and thus likely also δ^13^C values as they are less easily altered than δ^18^O _CO3 _values. This was taken as an indicator that even the bone and dentine samples may be reasonably well-preserved and can be interpreted to infer feeding ecology and habitat use of these ancient horses.

Several authors [42-44] suggest a high correlation between these two phases in some European and North American equids. In South America, the equation obtained for *Equus(Amerhippus) *from Argentina [45] was: δ^18^O _CO3 _= 16.74 δ^18^O _PO4 _+ 0.64; R^2 ^= 0.91. More recently, Sánchez et al. [8,32] also found that a significant relationship existed between the δ^18^O results in the carbonate and phosphate apatite phases (enamel, dentine, and bone) when they analyzed the oxygen isotopic composition of gomphotheres from several South American localities. The new data shows that δ^18^O _PO4 _and δ^18^O _CO3 _are highly correlated, with the latter being about 8.6 ‰ more positive than the former.

**Figure 3:**
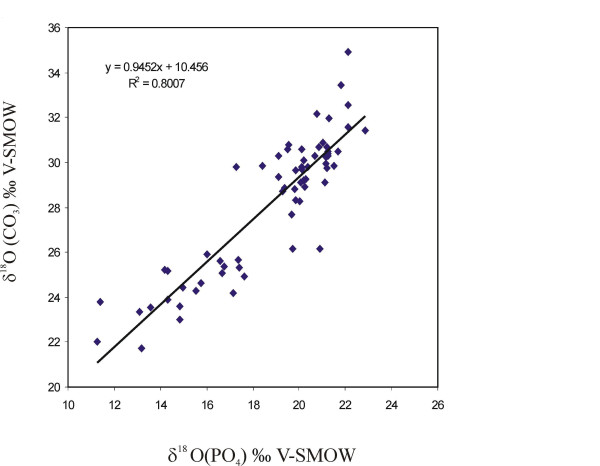
**Variation in δ**^**18**^**O _PO4 _and δ**^**18**^**O _CO3_**

We analyzed the δ^18^O _PO4 _and δ^18^O _CO3 _of the 26 samples for *Hippidion *and 41 samples for *Equus (Amerhippus) *in this way. The results of δ^18^O _PO4 _versus V-SMOW standard are reported in tables 1 and 2. The analytical variation for repeated analysis was 0.2‰. Each pair of δ^18^O values of teeth and bone belonged to the same individual, generally from the jaw or maxilla, the correlation being: δ^18^O _PO4 _= 0.9452 δ^18^O _CO3 _- 10.456; R^2 ^= 0.80. We also calculated the correlation between pairs of δ^18^O _PO4 _- δ^18^O _CO3 _values for the enamel, dentine, and bone, from the same individual. The results are plotted in Figure 3. The correlations between the different PO_4_-CO_3 _pairs are:

Enamel δ^18^O _PO4 _= 2.3371 δ^18^O _CO3 _- 19.095 R^2 ^= 0.97; Dentine δ^18^O _PO4 _= 1.2999 δ^18^O _CO3 _- 3.4034 R^2 ^= 0.389; and Bone δ^18^O _PO4 _= 1.1709 δ^18^O _CO3 _- 6.2038 R^2 ^= 0.88. In the first case it appears that the sample might have been modified by diagenesis because the carbonate results among the three skeletal phases (enamel, dentine, and bone) are different. In fact, the range of variation is 2.6‰ in the δ^18^O _PO4 _and 5.6‰ in the δ^18^O _CO3 _for the samples from the same specimen. Results of δ^13^C obtained from the bone are similar to those obtained from the dentine and are, in general, equal or more negative than results obtained from the enamel. We observed the same pattern in the δ^18^O results as we did in the δ^13^C results. The range of variation between the three skeletal phases (enamel, dentine, and bone) was small and we suppose that they have not been significantly altered by diagenesis. For these reasons, we consider these δ^13^C values as representative of each group of horses.

### Dietary Partitioning

**Figure 4:**
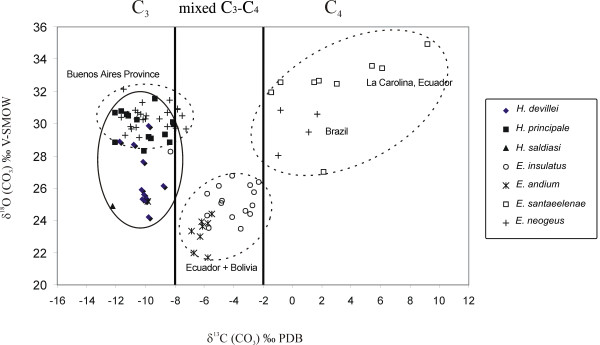
**Distribution of δ**^**18**^**O versus δ**^**13**^**C values of the South American equids**.

The carbon isotopic ratio of *Equus *(*Amerhippus*) and *Hippidion *remains indicate significant ecological differences (Figure 4). The *Hippidion *samples analyzed here are more homogeneous than the *Equus *(*Amerhippus*) samples, with a δ^13^C range between -12,9 and -8,0 ‰ (Table 1 and 2).

**Figure 5:**
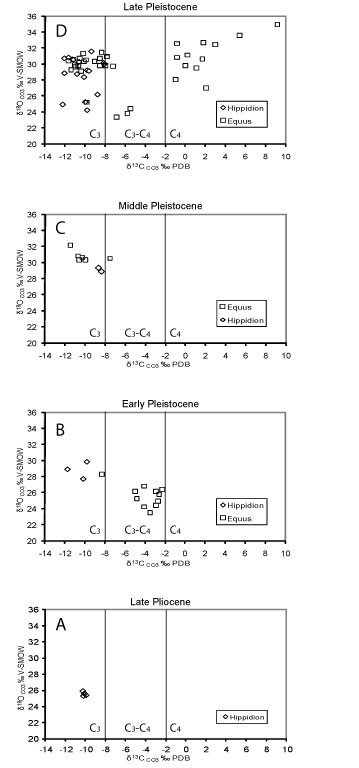
**Variation in δ**^**13**^**C and δ**^**18**^**O with time for South American *Hippidion *and *Equus***.

All the species of *Hippidion *were almost exclusively C_3 _feeders but some individuals from Bolivia and Argentina fall at the lower end of the mixed C_3_/C_4 _range. For instance, *H. principale *from the Eastern corridor (at sea level) and *H. devillei *from the Andes corridor yield similar δ^13^C values suggesting that they ate mainly C_3 _plants. The same pattern of dietary partitioning was obtained when comparisons were made between the same taxa at different latitudes (between 22°S and 52°S). From the upper Pliocene (*H. devillei *from Uquía locality) to the lower Pleistocene (*H. principale*, from the province of Buenos Aires and *H. devillei *from the Tarija locality) the dietary partitioning remains similar. The same pattern in dietary partitioning is observed throughout the Middle to Late Pleistocene (Figure 5) showing a predominance of C_3 _plants. Also, we did not find differences between *H. saldiasi *from the Ultima Esperanza in southern Patagonia and the other *Hippidion *species present at different localities across South America.

*Equus *species have predominantly been grazers, and as such, carbon isotopic values provide evidence of the C_3 _and C_4 _grasses. The carbon isotope data indicates that *Equus *(*Amerhippus*) shows three different patterns of dietary partitioning. Samples of *E*. (*A*.) *neogeus *from the province of Buenos Aires indicate a preference for C_3 _plants in the diet. The samples from Ecuador and Bolivia [*E*. (*A*.) *andium *and *E*. (*A*.) *insulatus*] show a preference for a diet of mixed C_3_-C_4 _plants, while those from La Carolina (sea level of Ecuador), Bolivia, and Brazil are mostly C_4 _feeders.

As mentioned before, a few outliers (e.g. δ^13^C values of 9,2; 6,1 and 5,4‰ from La Carolina) cannot be easily explained. These extremely high δ^13^C values (above 3‰) cannot be explained by consumption of C_4 _vegetation, which should impart an upper limit of about 3‰. These outliers could be the result from one of several possibilities, such as individuals living in costal peninsula areas of Ecuador during the time in which C_4 _grasses were abundant and may have produced δ^13^C values not observed in the modern ecosystem, or the sample presents taphonomic alteration. Specimen number V.3037 has a high variation between dentine, enamel, and bone (9,2; 1,5 and 6,1‰ respectively). MacFadden et al [36] obtained a δ^13^C value of 1,8‰ for the enamel of the same specimen but obtained 5,4‰ for the enamel of specimen number v.68 from the same locality. A more definitive explanation for these outliers must await the analysis of additional samples from these regions.

The δ^18^O _CO3 _results of the horse remains differ according to altitudinal and latitudinal distribution. There is a clear difference in the δ^18^O values obtained from the populations from the lower plains and those from high elevations. The lowest δ^18^O _CO3 _values are found in high elevation species from Ecuador and Bolivia [*E*. (*A*.) *insulatus *and *E*. (*A*.) *andium*] and range from 21,3 to 28,3‰ with an average of around 25‰. The *H. saldiasi *samples from southern Patagonia are also included in this group, their low δ^18^O values being caused by the effects of altitude and latitude. The second group is mostly represented by *E*. (*A*.) *neogeus*, *H. principale *and *H. devillei*. These species come from the province of Buenos Aires, Brazil, and Bolivia. The distribution values range from 27,7 to 32,1‰ with an average of around 30‰. The highest values come from the Carolina Peninsula in Ecuador, with δ^18^O _CO3 _maximum of 34,9‰.

## Discussion

### Areas of sympatry

As might be expected from ecological theory, finer-scale preliminary results from this study suggest feeding and niche differentiation within coexisting horses. *Hippidion *and *Equus (Amerhippus) *are sympatric in the same stratigraphic level in two localities: Tarija (22 °S) in Bolivia and the Pampas in Argentina (38 °S). Data from both localities suggest some difference in dietary partitioning. In a previous study, MacFadden and Shockey [34] presented carbon isotopic results from Tarija herbivores that, based on dental morphology, span the spectrum from presumed browsers (e.g., tapirs) to presumed grazers (e.g., horses) coexisting in the same localities. They suggested than the horses clearly occupied different dietary niches and can be separated using the carbon isotopes and hypsodonty index. *Hippidion *is the least hypsodont taxon and has the most negative mean δ^13^C value, whereas the relatively hypsodont *Equus *has the most positive mean δ^13^C value. As has been shown in another article [36], *Equus *is usually among the most grazing adapted mammalian herbivore in Pleistocene terrestrial ecosystems. This position in the herbivore community is similar at Tarija, whereas *Hippidion *are more adapted to the browsing end of the spectrum and *Equus *are primarily C_4 _grazers based on their carbon isotopic signature.

Our data suggests that the range of δ^13^C values for *H. devillei *and *H. principale *from Tarija falls in the low end of the mixed C_3_/C_4 _range (-10,8 to -6,6‰), whereas *E. (A.) insulatus *falls in the higher end (-8,3 to -2,3‰). There is some overlap with at least the two values of -8,3 and -6,6‰ indicating some differences in isotopic niche, with *H. devillei *consuming less grass than *E. (A.) insulatus *at this place and time [36].

In the data from Arroyo Tapalque (38 °S), one of the localities in the Pampas, the range of δ^13^C values for *H. principale *(-11,6 to -9,3‰) and *E. (A.) neogeus *(-8,5 to -7,9‰) do not overlap, but both are close that they suggest the same pattern of dietary partitioning as in Tarija.

If we look at the morphology, these taxa are very different. Several previous papers based on cranial and limb morphology associate these differences with browsing and grazing diet preference [e.g. [46]]. In general, the teeth of *Equus (Amerhippus) *are more hypsodont than *Hippidion*, and the enamel patterns are more complicated in *Equus (Amerhippus)*.

Another difference between sympatric species is body sizes. For instance, *H. principale *and *E*. (*A*.) *neogeus *are sympatric in the Pampas in Argentina. Both have a large body size, adapted to open habitat, but they differ in body mass (460 kg and 378 kg, respectively) [47]. The skulls of *E*. (*A*.) *neogeus *are big and show an enlarged preorbital and nasal region. The limb bones are large and robust, but more slender than in the other South American *Equus *species [3,4]. On the other hand, *H. principale *has a retracted nasal notch might signify some sort of proboscis. The skeleton is large and bulky, and the extremities are robust, mainly the metapodials and phalanges. It is the largest and strongest of the South American hippidiforms. These characteristics are classically associated with dietary and habitat preferences. The morphology indicated that *H. principale *may have been a browser but was able to live in open grasslands and that *E*. (*A*.) *neogeus *is the most hypsodont horse, and has the relatively straight muzzle characteristic of grazing horses [46].

### The effect of latitude

**Figure 6:**
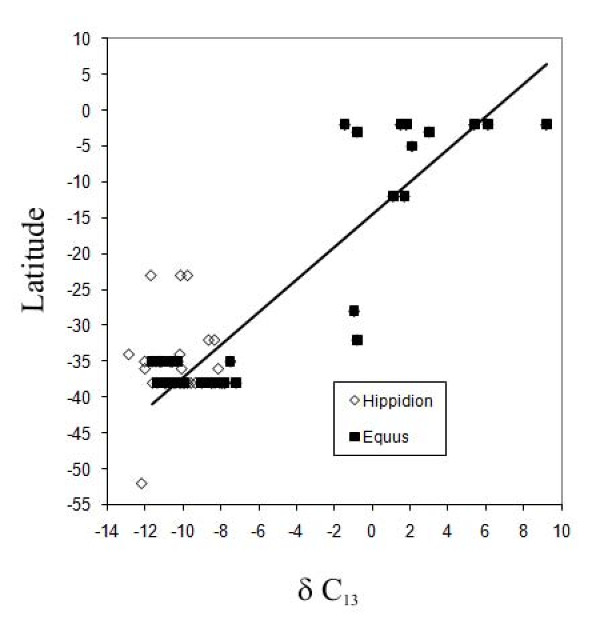
**Latitudinal distribution of δ**^**13**^**C values for the two species of South American equids**.

Some of the most fundamental patterns in global biogeography are those that are structured by the latitudinal gradients that extend from pole to equator (Figure 6). The proportion of C_3 _to C_4 _grasses in most modern ecosystems rises with increasing latitude. C_3 _grasses are predominant (>90%) in high-latitude steppes and prairies, whereas C_4 _grasses are predominant (>70-90%) in most low-elevation, and equatorial grasslands. The transition between C_3 _and C_4 _dominance in grasslands occurs at about 40-45° latitude in the Northern Hemisphere [48-50]. Exceptions to this general rule include grasses at high-elevations or in climates with cool-growing seasons (e.g. the Mediterranean basin), where the grasses are predominantly C_3 _regardless of latitude [50-52], and the occasional C_4 _grass species that are found in Arctic regions [53].

MacFadden et al. [36] used the Pleistocene distribution of *Equus (Amerhippus) *in the Americas to present a general δ^13^C gradient that seems to be symmetrical on either side of the Equator. They found that the isotopic transition between a full C_4 _signal and full C_3 _signal is observed at about 45°N in the northern hemisphere. Although there are considerably fewer data points in the southern hemisphere they [36] have found a signal of exclusively C_3 _feeders at around 35°S (province of Buenos Aires). A similar pattern in the Southern Hemisphere was found by Sánchez et al. [32] in the distribution of gomphotheres in South America. Samples of gomphotheres from the province of Buenos Aires, at around 39°S latitude show a mean δ^13^C value of -10,8‰, while samples from Chile (from several localities around 35° to 41°S) show a mean δ^13^C value of -12,3‰. This fact would confirm the existence of a latitudinal gradient for the Southern Hemisphere, and places the transition between a full C_3 _signal and mixed C_3_/C_4 _signal around 35° to 41°S.

The new data for the genus *Equus *(*Amerhippus*) analyzed in this paper are in agreement with the δ^13^C latitudinal pattern postulated by MacFadden et al. [36], while data for *Hippidion *also seem to follow a clear pattern. Samples of *Hippidion *show a boundary between an exclusively C_3 _to a mixed C_3_/C_4 _diet at Tarija (22°S), but in this case there is an effect of altitude combined with latitude. At the highest latitude specimens from Patagonia (52°S), the mean value is -12.2‰. The middle latitude samples, from the province of Buenos Aires (34°S), show δ^13^C values that range between -12,9 and -8,1‰, while the lowest latitude specimens, from Tarija (22°S), show δ^13^C values that range between -6,6 to -0,8‰.

### Altitudinal gradient

There are also significant differences between the δ^18^O _CO3 _values for the two genera, even when the range of δ^18^O values for high altitude and equatorial samples are taken into account (Table 4). This demonstrates the effects of an altitudinal gradient. The *Hippidion *specimens from the Andes corridor (Bolivia and northern Argentina) and the *Equus *(*Amerhippus*) specimens from Ecuador and Bolivia show the lowest values (between 21,7 and 28,3‰). On the other hand, samples from around 500 m of altitude (Bolivia, Ecuador, Brazil, Peru, and Argentina) present values that correspond with more temperate conditions (27,7 to 32,1‰). The effects of low altitude and latitude on δ^18^O _CO3 _may also explain the higher value obtained for the samples from La Carolina, Ecuador (sea level, latitude 2°S), where samples showed δ^18^O values ranging from 32 to 34,9‰.

**Table 4:** Results of parametric (t - test) and non-parametric (Wilcoxon Signed-Rank) tests.

		*t*-test	Nonparametric
Variable	Comparison groups	*p*	*p*
δ^13^C	A vs. B	**3,6E-10**	**1,17E-06**
δ^13^C	C vs. D	0,97	0,73
δ^13^C	E vs F	0,12	0,33
δ^13^C	G vs. H	0,15	0,15
δ^13^C	C vs. E	**2,2E-05**	**2,9E-04**
δ^13^C	D vs. F	**3,1E-07**	**9,8E-04**
δ^13^C	I vs. J	0,17	**0,04**
δ^18^O	A vs. B	0,85	0,93
δ^18^O	C vs. D	**0,01**	**0,02**
δ^18^O	E vs F	0,08	0,18
δ^18^O	G vs. H	**1,4E-12**	**1,2E-05**
δ^18^O	C vs. E	0,52	0,46
δ^18^O	D vs. F	0,08	0,09
δ^18^O	I vs. J	**0,01**	**0,03**

Results of tests performed on twelve possible paired comparisons between two genera of South American equids. The definition of groups is the same as for table 3. ***p***: significance level. ***p ***< 0.05 in bold.

We calculated a regression of δ^18^O _PO4 _values with altitude to quantify the effects of altitude. Bryant et al. [44] suggest a high correlation between δ^18^O _PO4 _and δ^18^O _CO3 _in Miocene North American equids. We found a good correlation between δ^18^O _PO4 _values and altitude. The equation obtained was: δ^18^O _PO4 _= -0.0016 altitude + 20.47 (R^2 ^= 0.63). We obtained an altitudinal gradient of -0.16 δ unit/100 meters.

An important point concerns altitudinal gradients. It seems that the horses living at high altitudes, 1866 up to 2780 m above sea level, and at low to middle latitude (2° to 22°S) still have a C_4 _component in their diet. This is not surprising at all given the distribution of modern C_4 _plants in the central Andes. At present, three C_4 _Amaranthaceae species occur at high elevations (>4000 m) where C_4 _plants are rarely observed and the altitude record reported for any confirmed C_4 _species worldwide is 4800 m for the grass *Muhlenbergia peruviana *[54]. However, this is not true for our specimens from 4000 m, which have a pure C_3 _diet. In this central Andean region, a clear altitudinal vegetation gradient is present. Subparamo (2000-3000 m) presents mosaics where shrubs and small trees which alternate with grasslands; and Paramo proper (3000 - 4100 m), is dominated by grasslands and shows patches of woody species which occur only in sheltered locations and along water streams. This altitudinal vegetation gradient changed during the Pleistocene. The treeless vegetation above the upper forest line was most widespread during glacial times, whereas it was limited to small areas on mountain tops during interglacial times [55]. The fossil pollen records show that such oscillations in patterns of plant distribution were repeated many times during the Pleistocene Ice Ages [56]. During the Last Glacial Maximum, when atmospheric *p*CO_2 _was reduced by some 50%, C_4 _plants dominated the Paramo vegetation, while only the highest mountain tops were covered by C_3 _grasses because of the low temperatures. Such small patches of C_4_-rich vegetation are probably relicts from the last ice age during which paramo vegetation was mainly composed of small tussocks and tufts of C_4 _grasses [57].

The change in altitudinal vegetation gradients during the Pleistocene is one of several possibilities to explain the C_4 _dietary component in horses living at high altitudes. Another alternative explanation might be that the horses partially fed at lower altitudes.

## Conclusions

Based on modern analogues, Pleistocene horses are inferred to be grazers but none of the grazing horses were interpreted as consumers of only C_4 _grasses. Our data shows that *Equus *(*Amerhippus*) had three different patterns of dietary partitioning. *E*. (*A*.) *neogeus *from the province Buenos Aires indicates a preference for C_3 _plants. *E*. (*A*.) *andium *from Ecuador and *E*. (*A*.) *insulatus *from Bolivia show a preference in a mixed diet of C_3_-C_4 _plants, while *E*. (*A*.) *santaeelenae *from La Carolina (sea level of Ecuador) and Brazil are mostly C_4 _feeders. These results confirm that ancient feeding ecology cannot always be inferred from dental morphology.

The record from South America suggests that *Hippidion *is in general a higher latitude taxon than *Equus *(*Amerhippus*). The highest latitude occurrence of *Hippidion *appears to be in southern Bolivia, in contrary to *Equus *(*Amerhippus*) where the occurrences are further north, and alone goes a long way in explaining the isotopic differences.

The data for *Hippidion *indicates a preference for C_3 _plants and mixed C_3_-C_4 _plants, but most of this data came from high altitude or latitude specimens. One possible, but unconfirmed explanation is that *Hippidion *were living in a "C_4 _World" and were browsers as indicated by their morphology, but more southern individuals were living at latitudes high enough to support C_3 _and C_4 _grasses.

The current study demonstrates the utility of using wide-ranging fossil mammals to explore latitudinal gradients and patterns of C_3_/C_4 _grass distribution and continental palaeotemperature during the Pleistocene. The carbon isotope composition of horses decreased as latitude increased. In *Equus *(*Amerhippus*) we found a change in signal between a mixed C_3_/C_4 _diet and a pure C_4 _diet around 32°S and a boundary between mixed diet and exclusively C_3 _signals at 35°S.

We also found that the horses living at high altitudes and at low to middle latitudes still have a C_4 _component in their diet, except for those specimens living at 4000 m, which have a pure C_3 _diet. The change in altitudinal vegetation gradients during the Pleistocene is one of several possibilities to explain the C_4 _dietary component in horses that lived at high altitudes. Another alternative explanation implies that the horses fed partially at lower altitudes.

## Abbreviations

(MLP): Museo de La Plata; (MACN): Museo Argentino de Ciencias Naturales "Bernardino Rivadavia", Buenos Aires; and (NCNAM): Museo de Ciencias Naturales y Antropológicas "Juan Cornelio Moyano", Mendoza, in Argentina; and (MEPN): Museo de la Escuela Politécnica Nacional of Quito, in Ecuador.

## Authors' contributions

Conceived and designed the experiments: JLP MTA. Performed the experiments: BS. Analyzed the data: BS. Wrote the paper: JLP BS. Intellectual support and editorial input: JLP MTA. All authors read and approved the final manuscript.

## References

[B1] Alberdi MT La familia Equidae, Gray, 1821 (Perissodactyla, Mammalia) en el Pleistoceno de Sudamérica IV Congreso Latinoamericano de Paleontología 1987 1 Santa Cruz de la Sierra, Bolivia 484 499

[B2] Alberdi MT Prado JL Review of the genus *Hippidion *Owen, 1869 (Mammalia; Perissodactyla) from the Pleistocene of South America Zoological Journal of the Linnean Society 1993 108 1 22 10.1111/j.1096-3642.1993.tb02559.x

[B3] Prado JL Alberdi MT A quantitative review of the horse *Equus *from South America Paleontology 1994 37 459 481

[B4] Alberdi MT Prado JL El registro de *Hippidion *Owen, 1869 y *Equus (Amerhippus) *Hoffstetter, 1950 (Mammalia, Perissodactyla) en América del Sur Ameghiniana 1992 29 265 284

[B5] MacFaddden BJ Fossil Horses. Systematics, Paleobiology, and Evolution of the Family Equidae 1992 Cambridge University Press

[B6] MacFadden BJ Cerling TE Mammalian herbivore communities, ancient feeding ecology, and carbon isotopes: a 10 million year sequence from the Neogene of Florida Journal of Vertebrate Paleontology 1996 16 103 115 10.1080/02724634.1996.10011288

[B7] Cerling TE Harris JM MacFadden BJ Leakey MG Quade J Eisenmann V Ehleringer JR Global vegetation change through the Miocene/Pliocene boundary Nature 1997 389 153 158 10.1038/38229

[B8] Sánchez B Prado JL Alberdi MT Ancient feeding ecology and extinction of Pleistocene Horses from Pampean Region (Argentina) Ameghiniana 2006 43 427 436

[B9] Bryant JD Luz B Froelich PN Oxygen isotopic composition of fossil horse tooth phosphate as a recorder of continental paleoclimate Palaeogeography Palaeoclimatology Palaeoecology 1994 107 303 316 10.1016/0031-0182(94)90102-3

[B10] Wang Y Cerling TE MacFadden BJ Fossil horses and carbon isotopes: new evidence for Cenozoic dietary, habitat, and ecosystem changes in North America Palaeogeography Palaeoclimatology Palaeoecology 1994 107 269 279 10.1016/0031-0182(94)90099-X

[B11] Cerling TE Harris JM MacFadden BJ Griffiths H Carbon isotopes, diets of North American equids, and the evolution of North American C4 grasslands Stable Isotopes 1998 BIOS Scientific Publishers Ldt, Oxford 363 379

[B12] MacFadden B Solounias N Cerling TE Ancient diets, ecology, and extinction of 5-million-year-old horses from Florida Science 1999 283 824 827 10.1126/science.283.5403.824 9933161

[B13] Passey BH Cerling TE Perkins ME Voorhies MR Harris JM Tucker ST Environmental change in the great plains: An isotopic record from fossil horses The Journal of Geology 2002 110 123 140 10.1086/338280

[B14] Domingo L Grimes ST Domingo MS Alberdi MT Paleoenvironmental conditions in the Spanish Miocene-Pliocene boundary: isotopic analyses of Hipparion dental enamel Naturwissenschaften 2009 10.1007/s00114-008-0500-y 19190888

[B15] Tütken T Vennemann TW Stable isotope ecology of Miocene mammals of Sandelzhausen, Germany Paläontologische Zeitschrift 2009 83 207 22621681415

[B16] Van Dam JA Reichart GJ Oxygen and carbon isotope signatures in late Neogene horse teeth from Spain and application as temperature and seasonality proxies Palaeogeography, Palaeoclimatology, Palaeoecology 2009 274 64 81 10.1016/j.palaeo.2008.12.022

[B17] Feranec RS Hadly EA Paytan A Stable isotopes reveal seasonal competition for resources between late Pleistocene bison (*Bison*) and horse (*Equus*) from Rancho La Brea, southern California Palaeogeography, Palaeoclimatology, Palaeoecology 2009 271 153 160 10.1016/j.palaeo.2008.10.005

[B18] Smith BN Epstein S Two categories of ^13^C/^12^C ratios for higher plants Plant Physiology 1971 47 380 384 10.1104/pp.47.3.380 16657626 PMC365873

[B19] Vogel JC Fuls A Ellis RP The geographical distribution of kranz grasses in South Africa South African Journal of Science 1978 74 209 215

[B20] Ehleringer JR Field CB Lin ZF Kuo CY Leaf carbon isotope and mineral composition in subtropical plants along an irradiance cline Oecologia 1986 70 520 526 10.1007/BF00379898 28311493

[B21] Ehleringer JR Sage RF Flanagan LB Pearcy RW Climatic change and evolution of C4 photosynthesis Trends in Ecology and Evolution 1991 6 95 99 10.1016/0169-5347(91)90183-X 21232434

[B22] Cerling TE Wang Y Quade J Expansion of C4 ecosystems as an indicator of global ecological change in the Late Miocene Nature 1993 361 344 45 10.1038/361344a0

[B23] Cerling TE Harris JM Carbon isotope fractionation between diet and bioapatite in ungulate mammals and implications for ecological and paleoecological studies Oecologia 1999 120 347 363 10.1007/s004420050868 28308012

[B24] Passey BH Cerling TE Schuster GT Robinson TF Roeder BL Ehleringer JR Inverse methods for estimating primary input signals from time-averaged isotope profiles Geochim Cosmochim Acta 2005 69 4101 4116 10.1016/j.gca.2004.12.002

[B25] Lee-Thorp JA Van der Merwe NJ Carbon isotope analysis of fossil bones apatite South African Journal of Science 1987 83 712 715

[B26] Quade J Cerling TE Barry JC Morgan ME Pilbeam DR Chivas AR Lee-Thorp JA van der Merwe A 16-Ma record of paleodiet using carbon and oxygen isotopes in fossil teeth from Pakistan Chemical Geology (Isotope Geoscience Section) 1992 94 183 192 10.1016/0168-9622(92)90011-X

[B27] Latorre C Quade J McIntosh WC The expansion of C4 grasses and global change in the late Miocene: Stable isotope evidence from the Americas Earth and Planetary Science Letters 1997 146 83 96 10.1016/S0012-821X(96)00231-2

[B28] MacFadden BJ Middle Pleistocene climate change recorded in fossil mammal teeth from Tarija, Bolivia, and upper limit of the Ensenadan Land-Mammal Age Quaternary Research 2000 54 121 131 10.1006/qres.2000.2146

[B29] MacFadden BJ Diet and habitat of toxodont megaherbivores (Mammalia, Notoungulata) from the late Quaternary of South and Central America Quaternary Research 2005 64 113 124 10.1016/j.yqres.2005.05.003

[B30] MacFadden BJ Higgins P Ancient ecology of 15-million-year-old browsing mammals within C3 plant communities from Panamá Oecologia 2004 140 169 182 10.1007/s00442-004-1571-x 15148598

[B31] MacFadden BJ Cerling TE Prado JL Cenozoic terrestrial ecosystem evolution in Argentina: evidence from carbon isotopes of fossil mammal teeth Palaios 1996 11 319 327 10.2307/3515242

[B32] Sánchez B Prado JL Alberdi MT Feeding ecology, dispersal, and extinction of South American Pleistocene gomphotheres (Gomphotheriidae, Proboscidea) Paleobiology 2004 30 146 161 10.1666/0094-8373(2004)030<0146:FEDAEO>2.0.CO;2

[B33] MacFadden BJ Wang Y Cerling TE Anaya F South American fossil mammals and carbon isotopes: A 25 million - year sequence from the Bolivian Andes Palaeogeography, Palaeoclimatology, Palaeoecology 1994 107 257 268 10.1016/0031-0182(94)90098-1

[B34] MacFadden BJ Shockey BJ Ancient feeding ecology and niche differentiation of Pleistocene mammalian herbivores from Tarija, Bolivia: morphological and isotopic evidence Paleobiology 1997 23 77 100

[B35] MacFadden BJ Cenozoic mammalian herbivores from the Americas: reconstructing ancient diets and terrestrial communities Annual Reviews Ecology and Systematic 2000 31 33 59 10.1146/annurev.ecolsys.31.1.33

[B36] MacFadden BJ Cerling TE Harris JM Prado JL Ancient latitudinal gradients of C_3_/C_4 _grasses interpreted from stable isotopes of New World Pleistocene horse (Equus) teeth Global Ecology and Biogeography 1999 8 137 149

[B37] MacFadden BJ Tale of two rhinos: isotopic ecology, paleodiet, and niche differentiation of *Aphelops *and *Teleoceras *from the Florida Neogene Paleobiology 1998 24 274 286

[B38] Alberdi MT Menegaz AN Prado JL Formas terminales de *Hippidion *(Mammalia, Perissodactyla) de los yacimientos del Pleistoceno tardío - Holoceno de la Patagonia (Argentina y Chile) Estudios Geológicos 1997 43 107 115

[B39] Alberdi MT Prado JL Miotti L *Hippidion saldiasi *Roth, 1899 (Mammalia, Perissodactyla) at the Piedra Museo Site (Patagonia): their implication for the regional economy and environmental reconstruction Journal of Archaeological Science 2001 28 411 419 10.1006/jasc.2000.0647

[B40] Iacumin P Bocherens H Mariotti A Longinelli A Oxygen isotope analyses of coexisting carbonate and phosphate in biogenic apatite: a way to monitor diagenetic alteration of bone phosphate? Earth and Planetary Science Letters 1996 142 1 6 10.1016/0012-821X(96)00093-3

[B41] Martin C Bentaleb I Kaandorp R Iacumin P Chatri K Intra-tooth study of modern rhinoceros enamel δ^18^O: Is the difference between phosphate and carbonate δ^18^O a sound diagenetic test? Palaeogeography, Palaeoclimatology, Palaeoecology 2008 266 183 189 10.1016/j.palaeo.2008.03.039

[B42] Sánchez-Chillón B Alberdi MT Leone G Bonadonna FP Stenni B Longinelli A Oxygen isotopic composition of fossil equid tooth and bone phosphate: an archive of difficult interpretation Palaeogeography, Palaeoclimatology, Palaeoecology 1994 107 317 328 10.1016/0031-0182(94)90103-1

[B43] Delgado A Iacumin P Stenni B Sánchez B Longinelli A Oxygen isotope variations of phosphate in mammalian bone and tooth enamel Geochimica et Cosmochimica Acta 1995 59 4299 4305 10.1016/0016-7037(95)00286-9

[B44] Bryant JD Froelich PN Showers WJ Genna BJ Biologic and climatic signals in the oxygen isotopic composition of Eocene-Oligocene equid enamel phosphate Palaeogeography, Palaeoclimatology, Palaeoecology 1996 126 75 89 10.1016/S0031-0182(96)00071-5

[B45] Sánchez-Chillón B Alberdi MT Meléndez G, Blasco MF, Pérez I Taphonomic modification of oxygen isotopic composition in some South American Quaternary mammal remains II Reunión de Tafonomía y Fosilización 1996 353 35611401071

[B46] Alberdi MT Prado JL Caballos fósiles de América del Sur. Una historia de tres millones de años 2004 INCUAPA Serie Monográfica

[B47] Alberdi MT Ortiz Jaureguizar O Prado JL Patterns of body size changes in fossil and living Equini (Perissodacyla) Biological Journal of the Linnean Society 1995 54 349 379

[B48] Ehleringer JR Cerling TE Helliker BK C_4 _photosynthesis, atmospheric CO_2_, and climate Oecologia 1997 112 285 299 10.1007/s004420050311 28307475

[B49] Epstein HE Lauenroth WK Burke IC Coffin DP Productivity patterns of C_3 _and C_4 _functional types in the U.S. Great Plains Ecology 1997 78 722 731

[B50] Tieszen LL Hein D Qvortrup SA Troughton HJ Imbamba SK Use of δ^13^C values to determine vegetation selectivity in East African herbivores Oecologia 1979 37 351 359 10.1007/BF00347911 28309221

[B51] Cavagnaro JB Distribution of C3 and C4 grasses at different altitudes in a temperate arid region of Argentina Oecologia 1988 76 273 277 10.1007/BF00379962 28312206

[B52] Cabido M Ateca N Astegiano ME Anton ME Distribution of C3 and C4 grasses along an altitudinal gradient in northern Argentina Journal of Biogeography 1997 24 197 204 10.1046/j.1365-2699.1997.00085.x

[B53] Schwarz AG Redman RE C4 grasses from the boreal forest region of Northwestern Canada Canadian Journal of Botany 1988 66 2424 2430 10.1139/b88-329

[B54] Ruthsatz B Hofmann U Die Verbreitung von C4-Pflanzen in den semiariden Anden NW-Argentiniens mit einem Beitrag zur Blattanatomie ausgewahlter Beispiele Phytocoenologia 1984 12 219 249

[B55] Van der Hammen T The Pleistocene changes of vegetation and climate in tropical South America Journal of Biogeography 1974 1 3 26 10.2307/3038066

[B56] Boom AM Marchant R Hooghiemstra H Sinninghe Damsté JS CO_2_- and temperature-controlled altitudinal shifts of C4- and C3-dominated grasslands allow reconstruction of palaeoatmosphere *p*CO2 Palaeogeography, Palaeoclimatology, Palaeoecology 2002 177 151 168 10.1016/S0031-0182(01)00357-1

[B57] Hooghiemstra H Van der Hammen T Quaternary Ice-Age dynamics in the Colombian Andes: developing an understanding of our legacy Phil. Trans. R. Soc. Lond 2004 359 173 181 10.1098/rstb.2003.1420 PMC1693313 15101574

[B58] Koch PL Tuross N Fogel ML The effects of sample treatment and diagenesis on the isotopic integrity of carbon in biogenic hydroxylapatite Journal of Archaeological Science 1997 24 417 429 10.1006/jasc.1996.0126

[B59] Koch PL Fisher DC Dettman D Oxygen isotope variation in the tusks of extinct proboscideans: a measure of season of death and seasonality Geology 1989 17 515 519 10.1130/0091-7613(1989)017<0515:OIVITT>2.3.CO;2

[B60] Tudge AP A method of analysis of oxygen isotopes in orthophosphate: its use in the measurement of paleotemperatures Geochimica et Cosmochimica Acta 1960 18 81 93 10.1016/0016-7037(60)90019-3

[B61] Longinelli A Oxygen isotopic composition of orthophosphate from shells of living marine organisms Nature 1965 207 716 718 10.1038/207716a0

